# Parental Separation and Cardiometabolic Risk Factors in Late Adolescence: A Cross-Cohort Comparison

**DOI:** 10.1093/aje/kwx007

**Published:** 2017-04-21

**Authors:** Ana Luiza Gonçalves Soares, Helen Gonçalves, Alicia Matijasevich, Maija Sequeira, George Davey Smith, Ana M. B. Menezes, Maria Cecília Assunção, Fernando C. Wehrmeister, Abigail Fraser, Laura D. Howe

**Keywords:** adolescents, ALSPAC, cardiometabolic risk factors, cohort studies, divorce, parental separation, Pelotas Birth Cohort

## Abstract

The aim of this study was to explore the association between parental separation during childhood (up to 18 years of age) and cardiometabolic risk factors (body mass index, fat mass index, blood pressure, physical activity, smoking, and alcohol consumption) in late adolescence using a cross-cohort comparison and to explore whether associations differ according to the age at which the parental separation occurred and the presence or absence of parental conflict prior to separation. Data from the Avon Longitudinal Study of Parents and Children (ALSPAC, United Kingdom) (1991–2011) and the 1993 Pelotas Birth Cohort (Brazil) (1993–2011) were used. The associations of parental separation with children's cardiometabolic risk factors were largely null. Higher odds of daily smoking were observed in both cohorts for those adolescents whose parents separated (for ALSPAC, odds ratio = 1.46; for Pelotas Birth Cohort, odds ratio = 1.98). Some additional associations were observed in the Pelotas Birth Cohort but were generally in the opposite direction to our a priori hypothesis: Parental separation was associated with lower blood pressure and fat mass index, and with more physical activity. No consistent differences were observed when analyses were stratified by child's age at parental separation or parental conflict.

There is growing recognition that adverse experiences in early life may have implications for health later in life. Although multiple types of adverse childhood experiences tend to occur together (1, 2), the magnitudes of the associations and the mechanistic pathways to health outcomes differ (3, 4). Parental divorce or separation is prevalent and can be a highly stressful event requiring adjustments for both parents and children; it may also engender several psychological, behavioral, and health problems (5). It has been associated with reduced income and lowered educational achievement in the offspring, which can negatively affect health, as well as influencing other health-related factors, such as social relationships, psychological functioning, and health behaviors such as alcohol use and smoking (5–8).

Although a substantial body of evidence has examined the long-term consequences of parental divorce/separation on mental health (9–14), potential implications for physical health, including cardiometabolic health, have received less attention. The evidence currently available suggests that children who experience parental divorce are at increased risk of cardiovascular disease mortality (8), stroke (15), and obesity (16). However, most of this evidence comes from studies carried out in adult populations in high-income countries, and few studies have assessed the timing of parental divorce, with one study suggesting that earlier parental separation is associated with more adverse adult health outcomes (17). Thus, identifying whether associations emerge during adolescence or differ according to the age at which parental separation occurs is important for evaluating the need for early interventions to avoid early morbidity and mortality.

Recent reviews of the literature highlight the role of parental or familial conflict as an important mechanism through which parental divorce may affect child outcomes (18–23), suggesting that chronic and acute stress caused by conflict may be at least as important as the act of divorce per se. It is possible that the health consequences of parental divorce differ depending on whether the child was exposed to parental conflict prior to their separation, but to our knowledge this has not been explored in studies to date.

In the present study, we used data from 2 cohort studies in contrasting settings (the United Kingdom and Brazil) to explore the association between parental separation up to the child's age of 18 years and cardiometabolic risk factors (body mass index (BMI), fat mass index (FMI), blood pressure, physical activity, smoking, and alcohol consumption) in late adolescence, and to explore whether the associations differ according to the age at which separation occurred and the presence or absence of parental relationship conflict.

## METHODS

### Research settings and study design

Data from the Avon Longitudinal Study of Parents and Children (ALSPAC, United Kingdom) and the 1993 Pelotas Birth Cohort (Brazil) were used. Using a cross-cohort comparison can improve causal inference: If associations are causal, we would expect them to be present in both cohorts, despite different confounding structures (24).

ALSPAC is a population-based longitudinal study that recruited pregnant women resident in the Avon area of the United Kingdom with an expected delivery date between April 1, 1991, and December 31, 1992 (25). The cohort consists of 14,775 live-born children (75.7% of the eligible live births) (26). The participants have been followed regularly through questionnaires and clinical assessments. At the 18-year follow-up, a total of 5,196 adolescents attended the clinical assessment (26). The ALSPAC website contains details of all the data that are available through a fully searchable data dictionary (http://www.bris.ac.uk/alspac/researchers/data-access/data-dictionary/).

The 1993 Pelotas Birth Cohort recruited all children born alive in hospitals in the urban area of the city of Pelotas, in Southern Brazil, between January 1, 1993, and December 31, 1993. Of the 5,265 births recorded, parents of 5,249 agreed to take part in the study (27). After baseline data collection, various subsamples were evaluated, and at 11 years of age the first attempt was made to locate all cohort members, achieving 87.5% follow-up (*n* = 4,452). At age 15 years, 85.7% were interviewed (*n* = 4,325), and at age 18 years, 81.3% (*n* = 4,106) of the original cohort was traced and interviewed (28). The questionnaires used for 1993 Pelotas Birth Cohort are available at (http://www.epidemio-ufpel.org.br/site/content/coorte_1993/).

### Measures

#### Exposure

Baseline relationship status was derived by asking whether the mother was married or living with a partner. Any divorce or separation reported after this by either the mother or the child in multiple questionnaires was considered to indicate parental separation. More details on how parental separation was assessed in each cohort are available in Web Appendix 1 (available at http://aje.oxfordjournals.org/).

In both cohorts, our analysis was therefore restricted to families where the parents were cohabiting at baseline. Separations of those mothers who began cohabiting after the baseline period were not considered due to the difficulty of ascertaining whether a later relationship was with the biological father or a stepfather and the possibility of multiple relationship formations and dissolutions. The age at parental separation was explored in both cohorts, and was categorized as being in early childhood (before age 5 years), late childhood (ages 5–9 years), or adolescence (ages 10–18 years).

#### Outcomes

BMI, FMI, systolic blood pressure (SBP), diastolic blood pressure (DBP), physical activity, daily smoking, and harmful use of alcohol were measured in both cohorts when the adolescents were on average 17.6 years of age in ALSPAC and 18.4 years of age in Pelotas, except for physical activity in ALSPAC, which was measured at a mean age of 15.5 years.

For both SBP and DBP, 2 arm measures were used. Physical activity was objectively measured using an accelerometer and recording minutes of moderate to vigorous physical activity (MVPA) based on valid days (29, 30). More details are available in Web Appendix 1.

Weight and height were measured at the clinic visit, and BMI was calculated by dividing weight (kg) by height (m) squared. Total body fat mass was obtained from dual-energy x-ray absorptiometry (DXA), and FMI was calculated dividing fat mass (kg) by height (m) squared. Daily smoking was used as a binary variable, based on whether the adolescent smoked every day, regardless of the number of cigarettes. Alcohol consumption was assessed by the Alcohol Use Disorders Identification Test (AUDIT) and classified using a cutoff at 8 points indicating harmful use (yes/no) (31). Both smoking and alcohol use information were obtained through a self-report questionnaire.

#### Covariates

Child's skin color (Pelotas), child's ethnicity (ALSPAC), family income, maternal age, maternal education/schooling, maternal and paternal smoking status (potential confounders), and interaction by parental relationship conflict were assessed in both cohorts. In ALSPAC, parental relationship conflict was based on maternal self-report assessed at 3 time points: child's infancy (assessed at age 22 months), early childhood (assessed at age 33 months), and mid-childhood (assessed at age 9 years). In Pelotas, parental relationship conflict was based on the adolescent's perception of his/her parents’ relationship assessed at 11-year follow-up. Full details of how these variables were assessed are provided in Web Appendix 1.

### Statistical analysis

Initially, descriptive statistics were generated for both cohorts, and the distributions of separation and outcome measures were explored according to number of socioeconomic and behavioral variables.

To minimize selection bias and increase efficiency, multiple imputation was used in both cohorts for those who had complete data on parental separation (participants considered to be eligible to this study). The imputation equations included the exposure, all outcomes, and all covariates and were stratified by sex. Web Tables 1 and 2 compare the distribution of imputed variables in the imputed data sets and the observed data (with no imputation) for ALSPAC and the Pelotas Birth Cohort, respectively, and show that distributions in imputed data sets were similar to observed distributions.

Unadjusted linear regression analyses were carried out, examining associations between parental separation and each continuous outcome measure. Logistic regression was used for the binary outcomes (daily smoking and harmful use of alcohol). Analyses of parental separation in relation to cardiometabolic risk factors were then adjusted for confounders as defined above. We a priori decided to look at interaction by age at parental separation and parental relationship conflict, and so we present stratified analyses for these variables. We tested for interaction by sex in the main model (using parental separation before age 18 years and each outcome measure, adjusted for confounders) and found evidence of interaction in Pelotas Birth Cohort for BMI (*P* = 0.026), FMI (*P* = 0.043), and SBP (*P* = 0.007). Thus, these results are presented stratified by sex. We also assessed the associations between parental conflict and each outcome of interest using linear/logistic regression, with and without adjustment for parental separation. To explore potential residual socioeconomic confounding, associations of socioeconomic position with parental separation and outcomes were assessed in both cohorts.

The analyses were performed using Stata, version 14.1 (StataCorp LP, College Station, Texas). All analyses were carried out using imputed data sets.

### Ethical approval

Ethical approval for the ALSPAC study was obtained from the ALSPAC Law and Ethics Committee and the local research ethics committee. The study protocol of the 1993 Pelotas Birth Cohort was approved by the Medical Ethics Committee of the Federal University of Pelotas, affiliated with the Brazilian Federal Medical Council. In both studies, the mothers (or caregivers) and adolescents provided written informed consent.

## RESULTS

In both cohorts, participants excluded from the analyses had lower family income, maternal schooling, and maternal age, and they were more likely to have parents with parental relationship conflict (Web Tables 3 and 4). In ALSPAC, those excluded from the analyses were more likely to be boys, to report white ethnicity, and to have parents who smoked, whereas in Pelotas they were more likely to report nonwhite skin color and to have nonsmoking parents.

In both cohorts, parental separation was inversely associated with family income, maternal education, and maternal age, and it was positively associated with parental relationship conflict (Table 1). In Pelotas, parental separation was also associated with nonwhite skin color, and in ALSPAC it was associated with parental smoking. In ALSPAC, 28.6% of the couples who were married or cohabiting at baseline had separated by the time their participating child was 18 years of age, and the highest proportion of separations (72%) occurred when the child was in adolescence. In Pelotas, the prevalence of parental separation was 36.5%, and 39% of the separations occurred during the child's adolescence (Table 1).

**Table 1.** Socioeconomic and Demographic Characteristics of Participants According to Experience of Parental Separation, Avon Longitudinal Study of Parents and Children (1991–2011) and 1993 Pelotas Birth Cohort (1993–2011)

Characteristic	ALSPAC			1993 Pelotas Birth Cohort		
	Not Separated (*n* = 3,626), %	Separated (*n* = 1,452), %	*P* Value^a^	Not separated (*n* = 2,272), %	Separated (*n* = 1,304), %	*P* Value^a^
Sex			0.088			0.458
Male	45.5	42.8		49.3	48.0	
Female	54.5	57.2		50.7	52.0	
Skin color/ethnicity			0.742			<0.001
White	94.6	94.5		71.2	64.3	
Black/mixed	5.4	5.5		28.8	35.7	
Family income (quintiles)			<0.001			<0.001
1 (poorer)	12.6	23.2		17.3	21.0	
2	18.9	21.0		22.6	25.2	
3	24.3	22.9		18.1	15.7	
4	19.9	14.7		19.7	21.0	
5 (better off)	24.3	18.2		22.3	17.1	
Maternal education			<0.001			
CSE/none	11.0	14.2				
Vocational	7.4	8.6				
O-level	33.8	36.9				
A-level	28.2	25.9				
Degree	19.6	14.4				
Maternal schooling, years						0.035
0–4				26.0	26.7	
5–8				46.3	49.2	
9–11				18.0	18.5	
≥12				9.7	5.6	
Maternal age, years			0.043			<0.001
<20	1.4	2.1		10.1	19.1	
20–34	85.8	86.7		76.0	72.6	
≥35	12.8	11.2		13.9	8.3	
Parental smoking			<0.001			0.524
No	63.9	45.7		27.7	28.7	
Yes	36.1	54.3		72.3	71.3	
Parental relationship conflict in early childhood			<0.001			
No	67.7	55.4				
Yes	32.3	44.6				
Parental relationship conflict in late childhood			<0.001			
No	65.9	51.2				
Yes	34.1	48.8				
Parental relationship conflict in adolescence			<0.001			<0.001
No	65.8	45.0		93.3	64.3	
Yes	34.2	55.0		6.7	35.7	
Parental separation by age of child, years			NA			NA
<5	NA	17.1		NA	33.2	
5–9	NA	10.5		NA	27.8	
10–18	NA	72.4		NA	39.0	

Abbreviations: ALSPAC, the Avon Longitudinal Study of Parents and Children; CSE, Certificate of Secondary Education; NA, not applicable.

^a^ Wald test for the difference between separated and not separated.

### Avon Longitudinal Study of Parents and Children

In unadjusted analysis, parental separation before the child's age 18 years was associated with higher BMI and FMI as well as higher odds of daily smoking (Table 2). After adjustment for confounders, most of these associations were no longer apparent, and parental separation before age 18 years was associated only with higher odds of offspring daily smoking (odds ratio (OR) = 1.46, 95% confidence interval (CI): 1.19, 1.78). The magnitude of this association was similar regardless of the age at which separation occurred (for separation <5 years of age, OR = 1.53; for separation at 5–9 years of age, OR = 1.41; and for separation at 10–18 years of age, OR = 1.44) (Table 3). Similar associations were also observed regardless of whether parental conflict was present (Web Tables 6 and 7). No associations were observed for any of the other outcomes either for the overall measure of parental separation or in the age- or conflict-stratified analyses. When the association between parental conflict and cardiometabolic outcomes was assessed, higher risks of offspring daily smoking (with conflict before age 5 years) and of alcohol use (with conflict before age 9 years) were found, even when accounting for parental separation (Web Table 8).

**Table 2.** Unadjusted and Adjusted^a^ Analysis of the Association Between Parental Separation and Cardiometabolic Risk Factors, Avon Longitudinal Study of Parents and Children (1991–2011) and 1993 Pelotas Cohort (1993–2011)

Risk Factor	ALSPAC (*n* = 5,078)						1993 Pelotas Cohort (*n* = 3,576)					
	Unadjusted			Adjusted			Unadjusted			Adjusted		
	β	OR	95% CI	β	OR	95% CI	β	OR	95% CI	β	OR	95% CI
*Continuous Outcomes*												
BMI^b,c^	0.28		0.02, 0.55	0.08		−0.19, 0.35						
Boys							−0.39		−0.81, 0.02	−0.41		−0.83, 0.01
Girls							0.30		−0.16, 0.76	0.24		−0.23, 0.70
FMI^b,c^	0.32		0.02, 0.62	0.04		−0.24, 0.31						
Boys							−0.75		−1.58, 0.08	−0.87		−1.70, −0.03
Girls							0.13		−0.30, 0.56	0.13		−0.32, 0.57
SBP, mm Hg^c^	−0.17		−0.88, 0.54	−0.12		−0.78, 0.53						
Boys							−2.20		−3.37, −1.04	−2.20		−3.39, −1.02
Girls							−0.12		−1.09, 0.86	−0.09		−1.07, 0.89
DBP, mm Hg	0.31		−0.10, 0.73	0.07		−0.35, 0.49	−0.93		−1.48, −0.38	−0.91		−1.46, −0.37
Physical activity (MVPA minutes/day)	0.06		−2.96, 3.09	0.06		−3.04, 3.16	4.43		1.10, 7.77	3.99		0.86, 7.13
*Binary Outcomes*												
Daily smoking		1.73	1.43, 2.10		1.46	1.19, 1.78		2.04	1.64, 2.54		1.98	1.58, 2.47
Harmful use of alcohol		1.03	0.87, 1.20		0.98	0.83, 1.15		1.35	1.16, 1.57		1.41	1.20, 1.66

Abbreviations: ALSPAC, the Avon Longitudinal Study of Parents and Children; BMI, body mass index; CI, confidence interval; DBP, diastolic blood pressure; FMI, fat mass index; MVPA, moderate to vigorous physical activity; OR, odds ratio; SBP, systolic blood pressure.

^a^ Adjusted for sex, skin color/ethnicity, family income, maternal education, maternal age, and parental smoking.

^b^ BMI was calculated as weight (kg)/height (m)^2^, and FMI was calculated as fat mass (kg)/height (m)^2^.

^c^*P* values for interaction by sex in Pelotas were 0.027 for BMI, 0.034 for FMI, and 0.006 for SBP; all other *P* values were >0.05.

**Table 3.** Multivariable^a^ Analysis of the Association Between Parental Separation and Cardiometabolic Risk Factors According to Age at Separation, Avon Longitudinal Study of Parents and Children (1991–2011) and 1993 Pelotas Cohort (1993–2011)

Risk Factor	Age at Separation, years								
	<5			5–9			10–18		
	β	OR	95% CI	β	OR	95% CI	β	OR	95% CI
*ALSPAC*									
BMI^b^	0.29		−0.26, 0.83	0.46		−0.26, 1.18	−0.02		−0.27, 1.18
FMI^b^	0.25		−0.32, 0.82	0.36		−0.35, 1.08	−0.06		−0.36, 0.24
SBP, mm Hg	0.17		−1.19, 1.54	0.73		−0.95, 2.41	−0.31		−1.05, 0.43
DBP, mm Hg	0.52		−0.39, 1.43	0.76		−0.43, 1.95	−0.13		−0.61, 0.35
Physical activity (MVPA minutes/day)	4.14		−4.83, 13.12	−0.35		−6.27, 5.57	−0.83		−3.94, 2.27
Binary outcomes									
Daily smoking		1.53	1.00, 2.33		1.41	0.87, 2.27		1.44	1.16, 1.80
Harmful use of alcohol		0.98	0.68, 1.41		0.85	0.57, 1.28		1.00	0.84, 1.36
*1993 Pelotas Birth Cohort*									
BMI^b,c^									
Boys	−0.76		−1.44, −0.09	−0.06		−0.75, 0.63	−0.40		−0.97, 0.17
Girls	0.60		−0.11, 1.32	0.15		−0.61, 0.91	−0.06		−0.74, 0.61
FMI^b,c^									
Boys	−1.23		−2.49, 0.04	−0.37		−1.69, 0.95	−0.94		−2.03, 0.16
Girls	0.43		−0.23, 1.09	0.10		−0.61, 0.81	−0.15		−0.78, 0.47
SBP, mm Hg^c^									
Boys	−2.83		−4.71, −0.96	−1.31		−3.25, 0.62	−2.34		−3.96, −0.73
Girls	0.18		−1.26, 1.63	0.14		−1.41, 1.70	−0.54		−1.94, 0.83
DBP, mm Hg	−0.97		−1.81, −0.14	−0.77		−1.65, 0.10	−0.96		−1.73, −0.18
Physical activity (MVPA minutes/day)	4.47		−0.26, 9.20	3.71		−2.10, 9.52	3.82		−0.65, 8.29
Binary outcomes									
Daily smoking		2.05	1.49, 2.82		2.06	1.46, 2.90		1.85	1.37, 2.51
Harmful use of alcohol		1.52	1.19, 1.93		1.50	1.16, 1.93		1.28	1.02, 1.61

Abbreviations: ALSPAC, the Avon Longitudinal Study of Parents and Children; BMI, body mass index; CI, confidence interval; DBP, diastolic blood pressure; FMI, fat mass index; MVPA, moderate to vigorous physical activity; OR, odds ratio; SBP, systolic blood pressure.

^a^ Adjusted for skin color/ethnicity, family income, maternal education, maternal age, and parental smoking. Unadjusted analysis is available in Web Table 5.

^b^ BMI was calculated as weight (kg)/height (m)^2^, and FMI was calculated as fat mass (kg)/height (m)^2^.

^c^*P* values for interaction by sex in Pelotas (from the model considering any separation before the child's age of 18 years) were 0.027 for BMI, 0.034 for FMI, and 0.006 for SBP; all other *P* values were >0.05.

### 1993 Pelotas Birth Cohort

Parental separation before age 18 years was associated with lower FMI and SBP (only in boys), lower DBP, higher MVPA, and greater odds of harmful alcohol use and daily smoking (Table 2) after adjustment for confounding. Those boys whose parents separated had 0.87 (95% CI: −1.70, −0.03) lower FMI and 2.20 mm Hg (95% CI: −3.39, −1.02) lower SBP than those whose parents did not, and adolescents whose parents separated before age 18 had 0.91 mm Hg (95% CI: −1.46, −0.37) lower DBP and performed 3.99 minutes/day (95% CI: 0.86, 7.13) more MVPA than those whose parents did not separate. Daily smoking was more frequent (OR = 1.98, 95% CI: 1.58, 2.47) among those whose parents separated, as was harmful use of alcohol (OR = 1.41, 95% CI: 1.20, 1.66). When analyses were stratified by age at parental separation (Table 3), the associations were strongest for BMI and separation at <5 years of age (boys only). Analyses stratified by age at separation showed weaker associations for separation at ages 5–9 years than for separation at <5 years of age or at ages 10–18 years with SBP and DBP, but associations were slightly weaker for separation at 10–18 years for smoking and harmful alcohol use. Stratification by parental conflict (Web Tables 6 and 7) did not alter most associations, with confidence intervals in the 2 groups (those exposed to conflict and those unexposed) overlapping for all outcomes, except SBP among boys in Pelotas (*P* for interaction = 0.038; all other *P* for interaction by conflict were >0.05). Parental conflict was associated with a higher odds ratio of daily smoking (conflict up to age 9 years) and a lower SBP, even when adjusting for parental separation (Web Table 8).

### Socioeconomic patterning of parental separation and cardiometabolic risk factors

Lower socioeconomic status (SES) was associated with parental separation in both cohorts. However, in ALSPAC, SES was inversely associated with BMI, SBP, and DBP, while in Pelotas SES was positively associated with boys’ BMI and inversely associated with girls’ BMI and was positively associated with MVPA, SBP, and DBP in both sexes (Web Table 9).

## DISCUSSION

This study described the relationship between parental separation and cardiometabolic risk factors in adolescents from 2 cohorts in different contexts. The association of parental separation with cardiometabolic outcomes was largely null apart from for smoking, where there was a strong association that was consistent across the 2 cohorts, even when controlling for SES. Some additional associations were observed in Pelotas. However, most of them were in the opposite direction from our a priori hypothesis, with parental separation being associated with slightly more favorable measures of cardiometabolic risk factors (lower SBP and FMI among boys, lower DBP, and higher practice of physical activity). The age at parental separation and presence of parental relationship conflict did not strongly or consistently influence the pattern of associations in these 2 cohorts.

The differences in the patterns of association observed across the cohorts may be due to differences in the confounding structures. Although low SES was associated with parental separation in both cohorts, we observed an association between low SES and higher MVPA (both sexes) and lower BMI and FMI (boys only) in Pelotas (in contrast to ALSPAC). Thus, residual confounding by socioeconomic factors could explain the negative associations of parental separation with FMI and MVPA found that were in Pelotas.

To our knowledge, no other study has assessed the association between parental separation and body composition, and some previous studies have shown that parental separation is associated with overweight in children (16, 32, 33), although less evidence is found in adolescents. A study carried out in Nordic countries that assessed the association between parental cohabitation and overweight in children aged 2 to 17 years reported an unadjusted odds ratio for overweight in those children whose parents lived separately that was higher in the age group 2–9 years than in the age group 10–17 years (in Iceland, for the youngest group, OR = 2.1, and for those aged 10–17 years, OR = 1.5) (34). It is possible that parental separation has some association with BMI in young children, but this association may not persist into adolescence, or the association may be context-specific. A cross-sectional study that investigated the association of childhood adversities, including parental separation, with blood pressure in adolescents aged 11–14 years found no evidence of an association. A study carried out in the United Kingdom using data from 2 British birth cohorts found that parental divorce before age 16 was associated with 25% higher chance of inactivity persistence and of activity reduction between the ages of 33 and 50 years (35). In contrast, our study found higher minutes of MVPA in those adolescents whose parents divorced before they were 18 years old but only in Pelotas.

Of the few sex differences observed in our study of the Pelotas Birth Cohort, the magnitudes of the associations for BMI, FMI, and SBP were larger among boys than among girls. This is in line with other studies that have suggested that male offspring are potentially more affected by paternal absence than are girls (36).

A higher risk of daily smoking was the only consistent association found between parental separation and cardiometabolic risk factors in both cohorts. Higher risk of harmful alcohol use was observed in Pelotas. The association of marital dissolutions with substance use has been explored by several studies, and previous studies support the association for cigarette smoking (14, 37–39) and alcohol use (40). These studies suggest the association can vary according to sex, with a higher risk observed among women (38), and according to age at divorce, finding a stronger association when parental divorce was experienced early in life (41). In our study, no interaction, either by sex or by age at separation, was found for the relationship of parental separation with daily smoking and harmful use of alcohol, although the association with smoking and alcohol in those whose parents separated when the child was aged 10–18 years was slightly lower in Pelotas compared with children whose parents separated at younger ages. The association between parental separation and smoking was consistent across the 2 cohorts and robust to adjustment for a wide range of confounders, including parental smoking. It is noteworthy that smoking is a key risk factor for cardiometabolic health, and so it is possible that associations between parental separation and other markers of health will emerge as these cohort participants get older. Smoking in adulthood has been found to mediate the association between parental divorce and health of adult children (17). Our findings suggest that this pattern can already be observed in adolescence.

The age at parental separation has been shown to be important in other studies when assessing the association of parental divorce/separation and health outcomes (14, 17, 41). In our study, for some outcomes the association was stronger when parental separation occurred in early childhood (e.g., BMI in boys in Pelotas), but the pattern was not consistent across cohorts or across outcomes. For some outcomes (e.g., SBP and DBP in Pelotas, and daily smoking in ALSPAC) the lack of association in those whose parents divorced when they were aged 5–9 years could be due to low power to detect the association, as parental separation was less frequent at this age.

Parental conflict before and during the divorce period is an important stressor for children, and it creates an aversive home environment (20), which could have a negative association with health outcomes. On the other hand, parental separation could potentially be less harmful to children if there is a high level of conflict within the household prior to separation. A review of the associations of marital conflict and dissolution with children's physical health showed that in some studies that assessed both marital structure and marital/familial functioning, the marital/familial functioning was more predictive of health outcomes than marital structure (23). Some evidence was found in our analyses for an association between parental conflict and higher risk of daily smoking and alcohol use, as well as lower SBP even when adjusting for parental separation. However, in our study, the presence of parental relationship conflict did not modify the association of parental separation on cardiometabolic risk factors.

### Strengths and limitations

This study used comparable data sets from 2 different contexts, with data collected using similar measures at similar ages. The outcomes were assessed using accurate measurements in both cohorts (e.g., dual-energy x-ray absorptiometry and accelerometer), although smoking and alcohol use were self-reported. However, the questionnaires gathering self-reports were administered in confidential conditions, which reduces underreporting.

The high rate of dropout and/or incomplete data, especially in ALSPAC, has to be considered, and—consistent with other cohorts—missing data and loss to follow-up were more common in those from socioeconomically deprived backgrounds (42). Although participants included in the analysis were less likely to have experienced parental separation than members of the full cohort overall, the associations between parental separation and cardiovascular outcomes were less likely to be affected by bias due to missing data (43). Furthermore, we used multiple imputation to minimize selection bias and to increase precision (44).

The different definition and time of assessment of parental relationship conflict between the 2 cohorts is also a limitation in this study. The measures in the 2 cohorts are likely to capture different aspects of relationship conflict, with the measure in ALSPAC being more specifically focused on conflict than the available measure in Pelotas (which could reflect additional aspects of relationship quality) and with reporting by the mother in ALSPAC but the child in Pelotas. In Pelotas, information on parental relationship conflict was assessed at the child's age of 11 years, so in this cohort we made the assumption that parental conflict preceded separation. Furthermore, the relationship children had with their parents before and after separation was not assessed, and the frequency and quality of contact with the parents are known to be important in the relationship between parental separation and health outcomes. The potential mediating and/or modifying role of remarriage was also not assessed here.

It is also important to highlight that parental separation may co-occur with other adversities, such as domestic violence, neglect, and substance abuse (1, 2). We did not adjust for other adversities in our analysis because data on the temporal relationship between adverse experiences was not available, and therefore other adversities could actually form part of the causal pathway of interest. However, it is likely that such adjustment would further attenuate any associations.

In conclusion, the present study showed little evidence of association between parental separation and cardiometabolic risk factors in adolescents, apart from for smoking. Age at parental separation and the presence of parental relationship conflict did not consistently modify the associations.

## Supplementary Material

Web Material
