# Supplementary material for: Parental Separation and Cardiometabolic Risk Factors in Late Adolescence: A Cross-Cohort Comparison
Source: Am J Epidemiol. 2017 Apr 21;185(10):898–906. doi: 10.1093/aje/kwx007 (PMC5430937; doi:10.1093/aje/kwx007)
Supplement: Web Material [file kwx007soareswebmaterialfinal.pdf]

Web Appendix 1

Assessment methods details

Details of assessment of parental separation

In ALSPAC, relationship status was derived at baseline (8 weeks gestation) from a questionnaire that asked whether the woman was married or living with a partner; for this analysis we created a binary variable of cohabitation (married or unmarried but cohabiting with the father of the child) versus all other relationship statuses. Any divorce or separation reported after by either the mother or the child in multiple questionnaires was considered to indicate parental separation.

In the 1993 Pelotas Cohort, mothers were asked in the perinatal questionnaire if they lived with a partner or husband; as in ALSPAC a binary variable for cohabitation versus any other relationship status was used. Adolescents reported at age 18 whether their parents had separated, and this was used as the measure of parental separation in this cohort.

Details of assessment of blood pressure and physical activity

In ALSPAC, for both SBP and DBP the mean of two right arm measures was used. PA was objectively measured using an Actigraph AM 7164 2.2 accelerometer, which the participants were requested to wear for 7 consecutive days during waking hours. Mean daily minutes of moderate-to-vigorous physical activity (MVPA) based on valid days was used[29].

In Pelotas, for both SBP and DBP the mean of two left arm measures was used. PA was objectively measured using a GENEActiv accelerometer, which the participants were requested to wear 24 hours a day for 5 to 8 days. The mean MVPA over valid assessment periods of at least 10 minutes was used[30].

Details of assessment of covariates included in the analyses

In ALSPAC, maternal age (years) was collected at 8 weeks gestation. Maternal education in 5 categories (CSE – Certificate of secondary education; Vocational degree; O level – ordinary level; A level – advanced level; University degree) was assessed at 32 weeks of pregnancy. Family income was measured at 33 and 47 months after delivery, and a mean family income was calculated; within-cohort quintiles of family income were generated from the full available sample. Information on ethnicity was reported at 12 years (white, black/mixed/other). Parental smoking was based on maternal and paternal self-report during pregnancy (8, 18 and 32 weeks) and 8 months after birth and those mothers and fathers who smoked at least one cigarette per day in any trimester of pregnancy or at 8 months after birth were classified as smokers. Relationship conflict was assessed based on four questions: (1) "How many arguments or disagreements have you and your partner had in the past three months? (none/ 1-3/ 4-7/ 8-13/ 14 or more)", "In the past 3 months, have any of these happened:" (2) "one of you walking out of the house", (3) "hitting or slapping partner", (4) "throwing or breaking things" (yes, I did this/ yes, he did this/ yes, we both did this/ no, not at all). A binary variable indicating relationship conflict was created, based on whether the couple had 8 or more disagreements or answered yes to any of the other questions. Relationship conflict was assessed at three time points: infancy (assessed at 22 months), early childhood (assessed at 33 months), and mid childhood (assessed at 9 years). Associations for separation <5 years were adjusted for the infancy measure, separation 5-9 years were adjusted for the early childhood measure, and 10-18 years were adjusted for the mid childhood measure.

In Pelotas, maternal age (years), maternal schooling (0-4 years; 5-8 years; 9-11 years; 12+ years), family income of the month prior to the delivery (quintiles), and parental smoking status were assessed at the perinatal visit. Quintiles of family income were generated

based on the full original sample. Parental smoking status was based on maternal self-report, and mothers and fathers who smoked at least one cigarette per day during any time of pregnancy were considered smokers. At 11 years old, skin color (white, non-white) and child-reported parental conflict were assessed. Parental relationship conflict was assessed by the statement "You think the relationship between your father and your mother is:", having as response options: excellent, very good, good, average or bad. A binary variable for relationship conflict was created, with conflict assumed to be present when the adolescent classified the parents' relationship as average or bad (the two lowest categories). Analyses were repeated with only bad parental relationship (6.6%) included as a measure of parental conflict and stronger associations between parental divorce and cardiometabolic outcomes were found, but overall conclusions were unaltered.

Web Table 1. Distribution of the Characteristics in the Observed and Imputed Data in the Avon Longitudinal Study of Parents and Children (ALSPAC) (1991-2011).

**Variable**	**Model used**	**N available**	**% imputed**	**Distribution: Mean (SE) for continuous variables, % for categorical variables**			
				Males		Females	
				Observed	Imputed	Observed	Imputed
Ethnicity	Logit	4,125	18.8				
White				94.5%	94.2%	95.1%	94.9%
Black/mixed				5.5%	5.8%	4.9%	5.1%
Maternal education	Ologit	4,948	2.6				
CSE/none				10.7%	10.8%	12.6%	12.8%
Vocational				7.7%	7.8%	7.6%	7.7%
O-level				33.4%	33.5%	35.7%	35.7%
A-level				28.9%	28.8%	26.6%	26.4%
Degree				19.3%	19.1%	17.5%	17.4%
Family income (quintiles)	Ologit	4,526	10.9				

1st (poorer)				13.7%	14.7%	15.3%	16.3%
2nd				18.7%	19.0%	19.4%	19.8%
3rd				25.0%	24.9%	23.4%	23.3%
4th				18.4%	18.0%	19.1%	18.7%
5th (better off)				24.2%	23.4%	22.8%	21.9%
Paternal smoking	Logit	5,047	0.1				
No				66.6%	66.3%	64.3%	64.1%
Yes				33.4%	33.7%	35.7%	35.9%
Harmful use of alcohol	Logit	3,532	30.4				
No				55.1%	55.3%	59.3%	59.3%
Yes				44.9%	44.7%	40.7%	40.7%
Diary smoking	Logit	3,846	24.3				
No				88.5%	88.0%	87.1%	86.8%
Yes				11.5%	12.0%	12.9%	13.2%
Maternal age (years)	Regress	4,823	5.0	29.46 (4.52)	29.41 (4.53)	29.09 (4.53)	29.04 (4.54)
BMI [a]	Regress	4,599	9.4	22.60 (3.88)	22.61 (3.88)	22.98 (4.41)	23.00 (4.41)

FMI [a]	Regress	4,409	13.2	4.35 (3.10)	4.45 (5.05)	7.89 (3.43)	7.94 (3.51)
SBP (mmHg)	Regress	4,264	16.0	124.27 (10.31)	124.33 (10.34)	113.98 (8.90)	114.00 (8.89)
DBP (mmHg)	Regress	4,264	16.0	62.99 (6.60)	63.03 (6.60)	64.53 (6.36)	64.54 (6.37)
PA (MVPA – min/day)	Regress	2,185	56.8	30.09 (20.26)	33.09 (33.65)	18.38 (15.21)	19.94 (24.18)

BMI: body mass index; FMI: fat mass index; SBP; systolic blood pressure; DBP: diastolic blood pressure; PA: physical activity; MVPA: moderate-to-vigorous physical activity

[a] BMI and FMI given as kg/m^2

Web Table 2. Distribution of the Characteristics in the Observed and Imputed Data in 1993 Pelotas Birth Cohort (1993-2011).

**Variable**	**Model used**	**N available**	**% imputed**	**Distribution: Mean (SE) for continuous variables, % for categorical variables**			
				Males		Females	
				Observed	Imputed	Observed	Imputed
Skin color	Logit	3,446	3.6				
White				70.0%	70.0%	67.2%	67.3%
Black/mixed				30.0%	30.0%	32.8%	32.7%
Maternal schooling (years)	Ologit	3,570	0.2				
0 – 4				25.8%	25.9%	26.7%	26.7%
5 – 8				47.5%	47.5%	47.2%	47.2%
9 – 11				18.0%	18.0%	18.3%	18.3%
12 +				8.7%	8.6%	7.8%	7.8%
Family income (quintiles)	Ologit	3,528	1.3				
1st (poorer)				18.8%	18.8%	18.6%	18.6%
2nd				24.7%	24.7%	22.4%	22.4%

3rd				17.1%	17.0%	17.4%	17.4%
4th				19.7%	19.8%	20.6%	20.6%
5th (better off)				19.7%	19.7%	21.0%	21.0%
Age at separation	Ologit	3,518	1.6				
Never separated				65.4%	64.2%	63.8%	63.0%
< 5 years				10.6%	10.9%	13.0%	13.3%
5 – 9 years				9.5%	9.8%	10.1%	10.4%
10 – 18 years				14.5%	15.1%	13.1%	13.3%
Paternal smoking	Logit	3,538	1.1				
No				49.0%	49.0%	50.4%	50.3%
Yes				51.0%	51.0%	49.6%	49.7%
Diary smoking	Logit	3,575	0.03				
No				89.0%	89.0%	90.7%	90.7%
Yes				11.0%	11.0%	9.3%	9.3%
Relationship conflict at adolescence	Logit	3,338	6.7				

No				83.0%	83.0%	82.5%	82.6%
Yes				17.0%	17.0%	17.5%	17.4%
BMI [a]	Regress	3,458	3.3	23.34 (4.22)	23.34 (4.22)	23.54 (4.77)	23.55 (4.77)
FMI [a]	Regress	3,351	6.3	4.25 (3.02)	4.84 (8.07)	8.41 (3.52)	8.59 (4.41)
SBP (mmHg)	Regress	3,471	2.9	130.73 (11.82)	130.72 (11.83)	115.05 (9.89)	115.06 (9.88)
DBP (mmHg)	Regress	3,471	2.9	71.00 (7.91)	71.00 (7.91)	69.41 (7.69)	69.40 (7.68)
PA (MVPA – min/day)	Regress	3,107	13.1	57.70 (44.83)	57.60 (48.01)	31.02 (30.20)	31.25 (32.09)

BMI: body mass index; FMI: fat mass index; SBP; systolic blood pressure; DBP: diastolic blood pressure; PA: physical activity; MVPA: moderate-to-vigorous physical activity

[a] BMI and FMI given as kg/m^2

Web Table 3. Socioeconomic and Demographic Characteristics of Participants with Complete Data Compared with Participants with Missing Data or Lost to Follow-up. The Avon Longitudinal Study of Parents and Children (ALSPAC Study, UK) (1991-2011).

**Variables**	**Participants included in the analysis %**	**Participants excluded from the analysis %**	**P value[a]**
Gender	N = 5,078	N = 9,061	< 0.001
Male	44.7	55.6	
Female	55.3	44.4	
Ethnicity	N = 4,125	N = 2,894	< 0.001
White	5.2	6.8	
Non-white	94.8	93.2	
Family income (quintiles)	N = 4,526	N = 5,464	< 0.001
1st (lowest)	14.5	25.8	
2nd	19.1	20.0	
3rd	24.1	21.0	
4th	18.8	16.2	
5th (highest)	23.5	17.0	
Mother’s education	N = 4,948	N = 7,514	< 0.001
CSE/none	11.7	25.8	
Vocational	7.7	11.3	
O-level	34.7	34.6	
A-level	27.6	19.0	
Degree	18.3	9.3	

Mother's age at birth (years)	N = 4,823	N = 6.987	< 0.001
< 20	1.5	5.0	
20 – 34	85.9	86.1	
35+	12.6	8.9	
Parental smoking	N = 5,078	N = 9,156	< 0.001
No	58.9	45.2	
Yes	41.1	54.8	
Parental separation	N = 5,078	N = 8,301	< 0.001
No	71.4	75.4	
Yes	28.6	24.6	
Relationship conflict at early childhood	N = 5,078	N = 9,576	< 0.001
No	64.2	38.9	
Yes	35.8	61.1	
Relationship conflict at late childhood	N = 5,078	N = 9,576	< 0.001
No	61.7	34.4	
Yes	38.3	65.6	
Relationship conflict at adolescence	N = 5,078	N = 9,576	< 0.001
No	59.9	24.3	
Yes	40.1	75.7	

[a] Chi-square test

Web Table 4. Socioeconomic and Demographic Characteristics of Participants with Complete Data Compared with Participants with Missing Data or Lost to Follow-up. 1993 Pelotas Birth Cohort, Brazil (1993-2011).

**Variables**	**Participants included in the analysis %**	**Participants excluded from the analysis %**	**P value[a]**
Gender	N = 3,576	N = 1,673	0.082
Male	48.8	51.4	
Female	51.2	48.6	
Skin color	N = 3,446	N = 975	< 0.001
White	68.5	60.7	
Non-white	31.5	39.3	
Family income at birth (quintiles)	N = 3,528	N = 1,609	0.004
1st (lowest)	18.7	23.1	
2nd	23.5	22.6	
3rd	17.3	17.4	
4th	20.1	18.1	
5th (highest)	20.4	18.8	
Mother's schooling (years)	N = 3,570	N = 1,673	0.001
0 to 4	26.3	31.7	
5 to 8	47.4	43.9	
9 to 11	18.1	16.4	
12 or more	8.2	8.0	
Mother's age at birth (years)	N = 3,576	N = 1,673	< 0.001

< 20	13.4	26.1	
20 – 34	74.8	64.7	
35+	11.8	9.2	
Parental smoking	N = 3,576	N = 1,673	< 0.001
No	28.5	33.5	
Yes	71.5	66.5	
Relationship conflict at adolescence	N = 3,338	N = 929	< 0.001
No	82.7	71.9	
Yes	17.3	28.1	

[a] Chi-square test

Web Table 5. Unadjusted Analysis of the Association Between Age at Parental Separation and Cardiometabolic Risk Factors in the Avon Longitudinal Study of Parents and Children (ALSPAC) (1991-2011) and 1993 Pelotas Cohort (1993-2011).

	**ALSPAC**			**Pelotas**		
	Separation < 5 y	Separation 5 – 9y	Separation 10 – 18y	Separation < 5 y	Separation 5 – 9y	Separation 10 – 18y
Continuous outcomes: Mean differences (95%CI) compared to children whose parents did not separate (null value = 0)						
BMI [a, b]	0.60 (0.04, 1.16)	0.81 (0.09, 1.53)	0.13 (-0.16, 0.43)			
Males				-0.68 (-1.35, -0.02)	-0.05 (-0.74, 0.64)	-0.41 (-0.98, 0.16)
Females				0.66 (-0.02, 1.33)	0.24 (-0.53, 1.02)	-0.02 (-0.69, 0.65)
FMI [a, b]	0.64 (0.02, 1.26)	0.72 (-0.07, 1.51)	0.19 (-0.15, 0.52)			
Males				-1.04 (-2.32, 0.25)	-0.26 (-1.63, 1.10)	-0.86 (-1.98, 0.26)
Females				0.44 (-1.20, 1.09)	0.11 (-0.60, 0.82)	-0.17 (-0.79, 0.46)
SBP (mmHg) [b]	0.10 (-1.37, 1.57)	1.14 (-0.69, 2.99)	-0.46 (-1.23, 0.30)			
Males				-2.77 (-4.64, -0.90)	-1.44 (-3.38, 0.50)	-2.29 (-3.91, -0.68)
Females				0.16 (-1.27, 1.59)	0.20 (-1.38, 1.79)	-0.64 (-2.06, 0.77)
DBP (mmHg)	0.79 (-0.14, 1.72)	0.99 (-0.19, 2.17)	0.01 (-0.47, 0.49)	-0.99 (-1.83, 0.16)	-0.81 (-1.70, 0.08)	-0.97 (-1.74, -0.20)
PA (MVPA	2.11 (-4.75, 8.97)	0.45 (-5.94, 6.84)	-1.50 (-4.42, 1.43)	3.25 (-1.90, 8.40)	4.27 (-1.60, 10.14)	5.57 (0.91, 10.22)

min/day)						
Binary outcomes: Odds ratios (95%CI) compared to children whose parents did not separate (null value = 1)						
Daily smoking	1.91 (1.22, 2.99)	1.77 (1.08, 2.88)	1.69 (1.35, 2.10)	2.05 (1.50, 2.81)	2.14 (1.53, 3.00)	1.96 (1.45, 2.64)
Harmful use of alcohol	1.03 (0.77, 1.38)	0.93 (0.64, 1.36)	1.04 (0.90, 1.21)	1.39 (1.11, 1.76)	1.43 (1.12, 1.83)	1.26 (1.00, 1.56)

ALSPAC: The Avon Longitudinal Study of Parents and Children; BMI: body mass index; FMI: fat mass index; SBP; systolic blood pressure; DBP: diastolic blood pressure; PA: physical activity; MVPA: moderate-to-vigorous physical activity

[a] BMI and FMI given as kg/m^2

[b] p-values for interaction by gender in Pelotas (from adjusted model considering any separation before 18 years) were 0.027 for BMI, 0.034 for FMI, and 0.006 for SBP; all other p-values were > 0.05

Web Table 6. Unadjusted Analysis of the Association Between Age at Parental Separation and Cardiometabolic Risk Factors in the Avon Longitudinal Study of Parents and Children (ALSPAC) (1991-2011) and 1993 Pelotas Cohort (1993-2011), According to Parental Relationship Conflict.

	**Separation < 5 y**		**Separation 5 – 9 y**		**Separation 10 – 18 y**	
	No conflict	Conflict	No conflict	Conflict	No conflict	Conflict
**ALSPAC**						
Continuous outcomes: Mean differences (95%CI) compared to children whose parents did not separate (null value = 0)						
BMI [a]	0.65 (-0.05, 1.35)	0.47 (-0.42, 1.35)	0.75 (-0.30, 1.80)	0.72 (-0.22, 1.66)	0.06 (-0.65, 0.48)	0.01 (-0.44, 0.46)
FMI [a]	0.59 (-0.06, 1.24)	0.64 (-0.72, 2.01)	0.71 (-0.62, 2.05)	0.62 (-0.27, 1.52)	-0.82 (-1.95, 0.31)	0.05 (-0.36, 0.45)
SBP (mmHg)	1.05 (-0.86, 2.97)	-1.29 (-3.58, 1.00)	1.17 (-1.56, 3.89)	0.82 (-1.73, 3.38)	-0.82 (-1.95, 0.31)	-0.28 (-1.41, 0.85)
DBP (mmHg)	0.92 (-0.28, 2.11)	0.56 (-0.94, 2.06)	0.16 (-1.53, 1.84)	1.48 (-0.17, 3.13)	-0.07 (-0.77, 0.63)	-0.05 (-0.74, 0.63)
PA (MVPA min/day)	2.70 (-5.06, 10.47)	1.41 (-9.28, 12.12)	-1.62 (-10.97, 7.71)	2.33 (-7.48, 12.14)	-1.74 (-5.71, 2.22)	-1.57 (-5.60, 2.46)
Binary outcomes: Odds ratios (95%CI) compared to children whose parents did not separate (null value = 1)						
Daily smoking	1.82 (1.04, 3.21)	1.89 (1.03, 3.46)	1.74 (0.83, 3.66)	1.64 (0.86, 3.13)	1.85 (1.37, 2.51)	1.42 (1.02, 1.98)
Harmful use of	1.13 (0.76, 1.68)	0.89 (0.55, 1.37)	0.83 (0.47, 1.45)	0.92 (0.55, 1.55)	0.99 (0.77, 1.26)	1.04 (0.84, 1.29)

alcohol						
**Pelotas Cohort**						
Continuous outcomes: Mean differences compared to children whose parents did not separate (null value = 0)						
BMI [a]						
Males	-0.93 (-1.80, -0.06)	0.002 (-1.33, 1.33)	-0.40 (-1.32, 0.53)	0.61 (-0.72, 1.95)	-0.22 (-0.86, 0.42)	-0.58 (-2.02, 0.86)
Females	0.41 (-0.47, 1.29)	0.59 (-1.01, 2.18)	0.10 (-0.92, 1.11)	0.00 (-1.62, 1.63)	-0.07 (-0.84, 0.70)	-0.30 (-2.10, 1.51)
FMI [a]						
Males	-1.23 (-2.96, 0.49)	-0.24 (-2.01, 1.53)	-0.56 (-2.44, 1.32)	0.62 (-1.22, 2.46)	-0.70 (-2.03, 0.62)	-0.98 (-2.88, 0.91)
Females	0.46 (-0.38, 1.30)	0.45 (-0.88, 1.78)	-0.07 (-1.02, 0.88)	0.34 (-0.99, 1.68)	-0.21 (-0.93, 0.51)	-0.02 (-1.52, 1.49)
SBP (mmHg)						
Males	-3.06 (-5.48, -0.64)	0.42 (-3.43, 4.28)	-2.81 (-5.47, -0.15)	3.02 (-0.76, 6.80) [b]	-2.19 (-4.00, -0.36)	-0.39 (-4.54, 3.76)
Females	0.14 (-1.62, 1.91)	1.66 (-1.74, 5.07)	0.78 (-1.37, 2.92)	1.04 (-2.44, 4.53)	-0.68 (-2.28, 0.91)	0.80 (-3.00, 4.61)
DBP (mmHg)	-0.87 (-1.91, 0.18)	-0.32 (-2.14, 1.51)	-0.67 (-1.86, 0.53)	-0.11 (-1.91, 1.69)	-0.95 (-1.82, -0.08)	-0.30 (-2.29, 1.69)
PA (MVPA min/day)	1.94 (-4.43, 8.32)	0.01 (-11.67, 11.70)	-1.18 (-8.66, 6.29)	5.53 (-6.70, 18.04)	6.69 (1.49, 11.90)	-2.44 (-16.05, 11.16)
Binary outcomes: Odds ratios compared to children whose parents did not separate (null value = 1)						

Daily smoking	1.85 (1.20, 2.83)	1.26 (0.67, 2.36)	1.65 (0.99, 2.75)	1.45 (0.78, 2.71)	2.14 (1.53, 2.99)	0.91 (0.43, 1.90) [c]
Harmful use of alcohol	1.54 (1.16, 2.05)	1.03 (0.62, 1.70)	1.48 (1.07, 2.05)	1.16 (0.70, 1.93)	1.35 (1.06, 1.73)	0.85 (0.48, 1.51)

ALSPAC: The Avon Longitudinal Study of Parents and Children; BMI: body mass index; FMI: fat mass index; SBP; systolic blood pressure; DBP: diastolic blood pressure; PA: physical activity; MVPA: moderate-to-vigorous physical activity

[a] BMI and FMI given as kg/m^2

[b] p-value for interaction between parental divorce and relationship conflict = 0.015

[c] p-value for interaction between parental divorce and relationship conflict = 0.038

Web Table 7. Adjusted [a] Analysis of the Association Between Age at Parental Separation and Cardiometabolic Risk Factors in the Avon Longitudinal Study of Parents and Children (ALSPAC) (1991-2011) and 1993 Pelotas Cohort (1993-2011), According to Parental Relationship Conflict.

	**Separation < 5 y**		**Separation 5 – 9 y**		**Separation 10 – 18 y**	
	No conflict	Conflict	No conflict	Conflict	No conflict	Conflict
**ALSPAC**						
Continuous outcomes: Mean differences (95%CI) compared to children whose parents did not separate (null value = 0)						
BMI [b]	0.39 (-0.31, 1.08)	0.07 (-0.80, 0.93)	0.29 (-0.79, 1.36)	0.51 (-0.42, 1.45)	-0.08 (-0.51, 0.36)	-0.04 (-0.49, 0.41)
FMI [b]	0.29 (-0.29, 0.88)	0.05 (-1.22, 1.33)	0.21 (-1.03, 1.45)	0.44 (-0.33, 1.13)	-0.12 (-0.61, 0.37)	-0.01 (-0.37, 0.35)
SBP (mmHg)	0.95 (-0.86, 2.76)	-1.04 (-3.20, 1.12)	0.84 (-1.67, 3.35)	0.33 (-1.99, 2.65)	-0.48 (-1.51, 0.55)	-0.28 (-1.34, 0.79)
DBP (mmHg)	0.68 (-0.48, 1.83)	0.30 (-1.18, 1.77)	-0.15 (-1.87, 1.57)	1.36 (-0.24, 2.96)	-0.26 (-0.97, 0.44)	-0.04 (-0.72, 0.65)
PA (MVPA min/day)	4.10 (-5.28, 13.47)	4.43 (-7.81, 16.67)	-0.89 (-10.19, 8.40)	0.52 (-7.60, 8.65)	-0.99 (-4.79, 2.81)	-0.79 (-5.30, 3.72)
Binary outcomes: Odds ratios (95%CI) compared to children whose parents did not separate (null value = 1)						
Daily smoking	1.50 (0.85, 2.65)	1.46 (0.76, 2.81)	1.31 (0.62, 2.79)	1.50 (0.77, 2.93)	1.52 (1.09, 2.11)	1.36 (0.97, 1.91)
Harmful use of	1.05 (0.69, 1.61)	0.86 (0.48, 1.53)	0.80 (0.44, 1.40)	0.83 (0.49, 1.42)	0.97 (0.76, 1.24)	1.00 (0.79, 1.27)

alcohol						
**Pelotas Cohort**						
Continuous outcomes: Mean differences (95%CI) compared to children whose parents did not separate (null value = 0)						
BMI [b]						
Males	-0.96 (-1.83, -0.09)	-0.47 (-1.82, 0.89)	-0.39 (-1.33, 0.55)	0.46 (-0.94, 1.87)	-0.24 (-1.33, 0.55)	-0.87 (-2.31, 0.59)
Females	0.41 (-0.53, 1.35)	0.60 (-1.03, 2.24)	0.03 (-0.97, 1.04)	-0.08 (-1.74, 1.57)	-0.05 (-0.81, 0.71)	-0.40 (-2.21, 1.41)
FMI [b]						
Males	-1.33 (-3.02, 0.36)	-0.94 (-2.76, 0.88)	-0.745(-2.57, 1.07)	0.40 (-1.41, 2.21)	-0.75 (-2.04, 0.54)	-1.26 (-3.10, 0.57)
Females	0.40 (-0.45, 1.26)	0.48 (-0.87, 1.84)	-0.15 (-1.10, 0.80)	0.35 (-1.09, 1.79)	-0.21 (-0.93, 0.52)	-0.04 (-1.57, 1.48)
SBP (mmHg)						
Males	-2.86 (-5.31, -0.42)	-0.53 (-4.31, 3.24)	-2.62 (-5.21, -0.02) [c]	2.96 (-0.78, 6.70) [c]	-2.15 (-3.99, -0.31)	-1.09 (-5.17, 2.98)
Females	0.07 (-1.72, 1.86)	2.06 (-1.50, 5.61)	0.77 (-1.38, 2.92)	1.17 (-2.39, 4.73)	-0.72 (-2.32, 0.87)	1.10 (-2.79, 4.98)
DBP (mmHg)	-0.79 (-1.87, 0.29)	-0.62 (-2.45, 1.22)	-0.61 (-1.79, 0.56)	-0.19 (-2.03, 1.64)	-0.93 (-1.80, -0.06)	-0.52 (-2.49, 1.46)
PA (MVPA min/day)	2.37 (-3.75, 8.49)	6.59 (-5.00, 18.17)	-1.51 (-8.75, 5.72)	8.68 (-3.80, 21.16)	4.91 (-0.18, 10.00)	-0.38 (-13.37, 12.62)
Binary outcomes: Odds ratios (95%CI) compared to children whose parents did not separate (null value = 1)						

Daily smoking	1.80 (1.17, 2.77)	1.53 (0.80, 2.91)	1.54 (0.91, 2.61)	1.63 (0.86, 3.09)	2.04 (1.45, 2.88)	0.99 (0.46, 2.11)
Harmful use of alcohol	1.72 (1.27, 2.32)	1.12 (0.65, 1.91)	1.53 (1.08, 2.15)	1.24 (0.73, 2.11)	1.36 (1.05, 1.75)	0.91 (0.50, 1.65)

ALSPAC: The Avon Longitudinal Study of Parents and Children; BMI: body mass index; FMI: fat mass index; SBP; systolic blood pressure; DBP: diastolic blood pressure; PA: physical activity; MVPA: moderate-to-vigorous physical activity

[a] Adjusted for sex, skin color, family income, maternal education maternal age, and parental smoking.

[b] BMI and FMI given as kg/m^2

[c] p-value for the interaction between parental separation and relationship conflict was 0.038; all other p-values for interaction by conflict > 0.05

Web Table 8. Multivariable Associations Between Parental Conflict and Cardiometabolic Risk Factors in the Avon Longitudinal Study of Parents and Children (ALSPAC) (1991-2011) and 1993 Pelotas Cohort (1993-2011).

	**Conflict before 5 y**		**Conflict before 5 – 9 y**		**Conflict before 10 – 18 y**	
	Model 1 [a]	Model 2 [b]	Model 1 [a]	Model 2 [b]	Model 1 [a]	Model 2 [b]
**ALSPAC**						
Continuous outcomes: Mean differences (95%CI) compared to children whose parents did not separate (null value = 0)						
BMI [c]	0.11 (-0.15, 0.36)	0.07 (-0.22, 0.37)	0.16 (-0.10, 0.42)	0.19 (-0.11, 0.48)	0.33 (0.08, 0.57)	0.28 (0.02, 0.54)
FMI [c]	0.11 (-0.15, 0.36)	0.08 (-0.24, 0.40)	0.00 (-0.26, 0.27)	0.05 (-0.27, 0.37)	0.12 (-0.13, 0.38)	0.07 (-0.20, 0.34)
SBP (mmHg)	0.15 (-0.48, 0.79)	-0.07 (-0.81, 0.67)	0.54 (-0.08, 1.16)	0.68 (-0.06, 1.42)	0.41 (-0.20, 1.03)	0.34 (-0.30, 0.98)
DBP (mmHg)	-0.09 (-0.52, 0.34)	-0.13 (-0.62, 0.37)	-0.06 (-0.50, 0.37)	0.18 (-0.33, 0.69)	0.15 (-0.29, 0.59)	0.08 (-0.37, 0.53)
PA (MVPA min/day)	-0.99 (-4.24, 2.27)	-1.10 (-5.02, 2.82)	-0.64 (-3.66, 2.37)	-0.95 (-4.49, 2.59)	-0.00 (-2.61, 2.61)	-0.11 (-2.76, 2.55)
Binary outcomes: Odds ratios (95%CI) compared to children whose parents did not separate (null value = 1)						
Daily smoking	1.32 (1.07, 1.64)	1.29 (1.00, 1.66)	1.11 (0.91, 1.36)	1.11 (0.87, 1.41)	1.21 (0.98, 1.51)	1.09 (0.86, 1.37)
Harmful use of alcohol	1.19 (1.03, 1.38)	1.22 (1.03, 1.46)	1.26 (1.09, 1.46)	1.33 (1.13, 1.58)	1.14 (0.99, 1.31)	1.15 (0.99, 1.33)

Pelotas Cohort

Continuous outcomes: Mean differences compared to children whose parents did not separate (null value = 0)						
BMI [c]	0.13 (-0.28, 0.54)	0.23 (-0.38, 0.85)	0.13 (-0.28, 0.54)	0.19 (-0.42, 0.81)	0.13 (-0.28, 0.54)	-0.12 (-0.71, 0.48)
FMI [c]	-0.09 (-0.66, 0.47)	-0.05 (-0.94, 0.84)	-0.09 (-0.66, 0.47)	0.13 (-0.80, 1.05)	-0.09 (-0.66, 0.47)	-0.25 (-1.13, 0.64)
SBP (mmHg)	-1.35 (-2.31, -0.37)	-1.72 (-3.18, -0.27)	-1.35 (-2.31, -0.37)	-1.75 (-3.25, -0.27)	-1.35 (-2.31, -0.37)	-2.14 (-3.58, -0.71)
DBP (mmHg)	-0.95 (-1.66, -0.25)	-0.80 (-1.84, 0.23)	-0.95 (-1.66, -0.25)	-0.85 (-1.94, 0.23)	-0.95 (-1.66, -0.25)	-0.77 (-1.82, 0.28)
PA (MVPA min/day)	5.03 (0.92, 9.15)	3.52 (-2.55, 9.59)	5.03 (0.92, 9.15)	6.01 (-0.45, 12.48)	5.03 (0.92, 9.15)	0.02 (-6.15, 6.20)
Binary outcomes: Odds ratios compared to children whose parents did not separate (null value = 1)						
Daily smoking	1.91 (1.48, 2.47)	1.71 (1.17, 2.51)	1.91 (1.48, 2.47)	1.91 (1.29, 2.84)	1.91 (1.48, 2.47)	1.32 (0.88, 1.99)
Harmful use of alcohol	1.17 (0.95, 1.43)	1.00 (0.74, 1.37)	1.17 (0.95, 1.43)	1.13 (0.83, 1.56)	1.17 (0.95, 1.43)	1.05 (0.77, 1.44)

ALSPAC: The Avon Longitudinal Study of Parents and Children; BMI: body mass index; FMI: fat mass index; SBP; systolic blood pressure; DBP: diastolic blood pressure; PA: physical activity; MVPA: moderate-to-vigorous physical activity

[a] Model 1: Adjusted for sex, skin color, family income, maternal education maternal age, and parental smoking

[b] Model 2: Model 1 + parental separation after parental conflict (separation < 5, separation 5-9 years or separation 10-18 years)

[c] BMI and FMI given as kg/m^2

Web Table 9. Unadjusted Associations Between Family Income and Cardiometabolic Risk Factors in the Avon Longitudinal Study of Parents and Children (ALSPAC) (1991-2011) and 1993 Pelotas Cohort (1993-2011) (Mean Difference & 95%CIs).

	**BMI [a]**	**FMI [a]**	**SBP (mmHg)**	**DBP (mmHg)**	**PA (MVPA min/day)**
**ALSPAC**					
Family income (per quintile change)					
Males	-0.12 (-0.25, 0.00)	-0.21 (-0.38, -0.05)	-0.04 (-0.43, 0.35)	-0.30 (-0.54, -0.07)	-1.53 (-3.52, 0.46)
Females	-0.31 (-0.44, -0.18)	-0.28 (-0.38, -0.18)	-0.35 (-0.62, -0.09)	-0.36 (-0.56, -0.16)	0.17 (-0.71, 1.06)
**Pelotas**					
Family income (per quintile change)					
Males	0.38 (0.24, 0.52)	0.29 (0.01, 0.56)	0.29 (-0.11, 0.69)	0.17 (-0.01, 0.43)	-5.62 (-7.48, -3.76)
Females	-0.11 (-0.28, 0.04)	0.02 (-0.13, 0.16)	-0.04 (-0.37, 0.29)	0.18 (-0.07, 0.44)	-1.90 (-3.08, -0.73)

ALSPAC: The Avon Longitudinal Study of Parents and Children; BMI: body mass index; FMI: fat mass index; SBP; systolic blood pressure; DBP: diastolic blood pressure; PA: physical activity; MVPA: moderate-to-vigorous physical activity

[a] BMI and FMI given as kg/m^2
